# Extracellular Vesicles from Canine Mesenchymal Stem Cells—Isolation, Characterization and miRNA Definition Following Interleukin-1ß and Shockwave Treatment

**DOI:** 10.3390/ani16121872

**Published:** 2026-06-17

**Authors:** Michele C. Klymiuk, Mohamed I. Elashry, Manuela Heimann, Kathrin Wolf-Hofmann, Susanne Schubert-Porth, Stefan Arnhold

**Affiliations:** Institute of Veterinary-Anatomy, -Histology and -Embryology, Faculty of Veterinary Medicine, Justus-Liebig-University Giessen, Frankfurter Strasse 98, 35392 Giessen, Germany; mohammed.elashry@vetmed.uni-giessen.de (M.I.E.); manuela.heimann@vetmed.uni-giessen.de (M.H.); kathrin.wolf@vetmed.uni-giessen.de (K.W.-H.); susanne.schubert-porth@vetmed.uni-giessen.de (S.S.-P.); stefan.arnhold@vetmed.uni-giessen.de (S.A.)

**Keywords:** MSC, EVs, NGS, miRNA, osteoarthritis, damage model, canine

## Abstract

Extracellular vesicles (EVs) have become a key area of research, as scientists study their role in various biological processes. These vesicles appear to play a key role in the use of mesenchymal signaling cells (MSCs, formerly known as mesenchymal stem cells) to treat various diseases, such as osteoarthritis (OA), and other degenerative conditions. In our experiments, we examined EVs formed by canine mesenchymal signaling cells (MSCs) to identify them according to current guidelines and define their content, particularly the microRNA (miRNA) they contain, for future research projects. After obtaining the EVs, we demonstrated via Western blotting and transmission electron microscopy that the nanoparticles visible in the nanotracking analysis were positive for CD9 and ALIX and positive for CD9 and CD81, respectively. Markers for nanoparticles that do not represent extracellular particles—tested here as cytochrome C for mitochondrial particles and histones for nuclear particles—were negative. Finally, we detected a total of 85 different miRNAs in the negative controls. To determine the potential influence of various cell stimulations intended to induce osteoarthritis (e.g., interleukin-1β stimulation) or a possible treatment (e.g., shockwave therapy), or the influence of ITS prior to extracellular vesicle extraction, we detected a total of 208 different miRNAs. These results demonstrate how canine EVs from MSCs can be detected in vitro and how the EVs’ miRNA profile changes after stimulation of the producing cells. This information may provide valuable insight into the understanding and treatment of osteoarthritis. Additionally, we demonstrated that using ITS instead of FCS to produce EVs should be reconsidered due to the significant change in miRNA expression levels.

## Simple Summary

Extracellular vesicles (EVs) play a central role in cell-to-cell communication and are becoming increasingly important in regenerative medicine. In this study, we isolated and characterized EVs from canine mesenchymal stem cells to evaluate their potential for future research. Nanotracking analysis revealed the particles’ typical size distribution, and the particles tested positive for the EV markers CD9, CD81, and ALIX but negative for the mitochondrial marker cytochrome C and the nuclear marker histone. A total of 85 different microRNAs (miRNAs) were detected in the untreated control samples. After stimulation with interleukin-1β, extracorporeal shockwave therapy, and ITS supplementation, 208 different miRNAs were identified. These changes in the miRNA profile suggest that the type of cell stimulation affects the molecular composition of the secreted EVs. These results provide valuable insight into extracellular vesicles derived from canine mesenchymal stem cells for future research.

## 1. Introduction

Extracellular vesicles (EVs) are nanoscale, membrane-bound particles ranging from 30 nm to 1 µm in size that are secreted by most cell types. They serve as a means of communication between cells and can carry various types of information, including proteins, nucleic acids, carbohydrates, and lipids [1]. These capabilities open up a wide range of possibilities for basic research and therapeutic applications. In this context, the formation of EVs from mesenchymal stem cells is of particular interest [2,3]. Mesenchymal stem cells are a known therapeutic agent for various degenerative diseases, such as osteoarthritis [4]. Contrary to earlier assumptions, it is well known that their main task is not to differentiate into cells of the desired tissue to replace defective cell formations. Rather, they play a mediating role. For this reason, Caplan (2017) recommends referring to them as medicinal signaling cells instead of mesenchymal stem cells [5]. Recently, EVs have emerged from the MSC secretome as special messengers. Using MSC-derived EVs has several advantages over transplanting the MSCs themselves. Cultivating autologous MSCs is significantly slower and less consistent than this method. It is also more difficult to ensure consistent quality and reduce the risk of side effects when using living cells [4]. Therefore, investigating the EVs formed by mesenchymal stem cells (MSCs) is essential to further establishing their mode of action and potential therapeutic use. One possible application is the treatment of canine osteoarthritis [6]. This disease is particularly significant for aging humans [7], dogs [6] and horses [8], and it has only been treated symptomatically thus far [9,10,11], as there are no effective alternatives, or the alternatives are difficult to apply due to the current legal situation regarding veterinary usage. In particular, producing MSCs for personalized canine medicine is only possible in a few facilities and is subject to many restrictions. Conversely, synthetic or cell-derived products could be a solution. For example, cell-derived EVs with their special miRNA cargo could be used.

MicroRNAs are short, single-stranded RNAs consisting of 18–24 nucleotides. Although they do not code, they can intervene in gene regulation at the post-transcriptional level. These short RNA fragments can profoundly influence cell function, including immunomodulation, differentiation, and proliferation. Since changes in cell metabolism lead to significant changes in expression patterns, miRNAs are being intensively researched as disease markers for many cancer types, including lung and breast tumors, as well as osteosarcomas, based on the concept of a liquid biopsy. Research in dogs has mainly focused on the expression of miRNAs in the aforementioned diseases [12].

Our work aims to isolate extracellular vesicles (EVs) from canine mesenchymal stromal cells (MSCs) and clearly demonstrate that no cellular components that could interfere with the isolation process, such as nuclear debris or mitochondrial components, were co-purified.

Additionally, we will characterize the microRNA (miRNA) content in the EVs. To accomplish this, we will examine not only native EVs, but also those released by MSCs that have been pretreated with interleukin-1 beta (IL-1β), shockwave therapy, or a serum-free medium with the addition of insulin, transferrin, and selenium (ITS). This research will lay the groundwork for further studies on the influence of miRNAs in an osteoarthritis model.

This research may be applicable to human medicine because many of the underlying biological processes of OA development and progression in dogs and humans are comparable [13]. The shared living environment and spontaneous onset of the disease, in addition to the pathophysiological foundations, are key factors that make dogs particularly suitable models for human osteoarthritis [14].

## 2. Materials and Methods

### 2.1. Obtaining AdMSCs from Canine Donors

Fat samples were obtained intraoperatively from dogs undergoing surgery for a non-inflammatory, non-infectious condition to obtain adipose-derived MSCs (AdMSCs). The adipose tissue removed during surgery to improve visualization was used for this purpose. The competent authority authorized further use of the adipose tissue (registration no. V 54-19 c 20 15 h 02 GI 18/1 kTV 1/2018).

The subsequent procedure has been published elsewhere [15]. The obtained sample was cut into pieces approximately the size of a pea. The pieces were then washed several times with PBS and incubated for one hour at 37 °C on a temperature-controlled shaker with 1 mg/mL of collagenase type 1. Then, the sample was centrifuged at 240× *g* for five minutes at room temperature and filtered through a 70 µm strainer. The filtrate was placed in a standard cell culture medium consisting of DMEM low glucose (order no. 11564446, Thermo Fisher Scientific, Dreieich, Germany), 10% FCS (batch no. 201004, PAN, Aidenbach, Germany), and 1% antibiotics (Penicillin–Streptomycin, order no. 11548876, Fisher Scientific, Dreieich, Germany) in 25 cm^2^ flasks (EasYFlasks™, Nunclon™Δ, 734-2066, VWR, Darmstadt, Germany) in an incubator at 37 °C with 5% CO_2_ and 95% relative humidity. The cells were propagated and then frozen in passage 2 for use in passage 3 in later experiments.

### 2.2. AdMSC Cultivation and Differentiation

Canine adipose-derived mesenchymal stem cells (AdMSCs) were cultured in standard cell culture medium (DMEM low glucose (LG) with 10% fetal calf serum (FCS) and 1% antibiotics). To verify the stem cell characteristics according to the International Society for Cellular Therapy (ISCT) standards [16], the following investigations were performed: growth with plastic adherence and differentiation into three lineages: chondrogenic, adipogenic, and osteogenic. Additionally, we carried out flow cytometric analysis of canine AdMSCs for the positive markers CD44 and CD90 and the negative markers CD45 and MHC II. These methods for validating AdMSC properties are routinely performed in our laboratory. These methods have already been published by our group and are therefore not described in full detail here [17,18,19].

Osteogenic and adipogenic differentiation was performed in 24-well plates (order number 734-2325, VWR, Germany) over a period of two weeks in the respective medium: The osteogenic medium consisted of DMEM LG, 5% FCS, 1% antibiotics, 0.1 µM dexamethasone, 10 mM β-glycerophosphate, and 60 µM ascorbic acid, while the adipogenic medium consisted of DMEM LG, 5% FCS, 1% antibiotics, 1% insulin–transferrin–selenite solution (ITS, order number ITS-H, Capricorn, Ebsdorfergrund, Germany), 1 µM dexamethasone, and 5 µM rosiglitazone. Chondrogenic differentiation was performed in a pellet culture over a period of two weeks. To achieve this, 0.3 million cells were added to an anti-adhesive, U-bottom 96-well plate (order no. 734-2782, VWR, Germany) and centrifuged at 120× *g* for five minutes to form small cell pellets. The cells were then incubated in a medium consisting of DMEM LG, 1% antibiotics, 1% ITS, 0.1 µM dexamethasone, 0.9 mM sodium pyruvate, 0.17 mM ascorbic acid, 0.35 mM proline, and 10 ng/mL TGFβ to induce differentiation.

After the incubation period, alizarin red staining was used to detect osteogenic differentiation, oil red O staining was used to detect adipogenic differentiation, and alcian blue staining was used to detect chondrogenic differentiation, following fixation and sectioning of the pellets [19].

Additionally, flow cytometric analysis was performed on the stem cell-specific positive markers CD90 (clone 5E10) and CD44 (clone IM7), as well as the negative markers MHC II (monoclonal antibody clone CVS20) and CD45 (clone UCHL1) [17,18,19].

### 2.3. AdMSC Treatment

#### 2.3.1. Interleukin 1ß Treatment

AdMSCs at approximately 80% confluence were incubated in a 75 cm^2^ cell culture flask containing DMEM LG, 1% antibiotics, and 1% exosome-depleted fetal calf serum (Exo-FCS, order no. FBS-ED-12G, Capricorn, Ebsdorfergrund, Germany) for three days, supplemented with interleukin 1β (IL-1β, order no. 200-01B, Thermo Fisher Scientific, Dreieich, Germany). For this purpose 10 µg/mL of IL-1β was added to the cell culture medium daily.

#### 2.3.2. Shockwave Treatment

Shockwave treatment was performed using the FB12 G5 transducer (Richard Wolf GmbH, Knittlingen, Germany) on the extracorporeal shockwave device (Piezovet 100 Plus, Richard Wolf GmbH, Knittlingen, Germany). The focus volume was 59 mm^3^ with an energy flux density of 1.1 mJ/mm^2^ and a pressure of 122 MPa. These results were achieved using treatment level 20, based on the manufacturer’s specifications.

For this treatment, the cells must be in suspension in order to concentrate them in the focus volume. The cells obtained after passage 2 were adjusted to a concentration of 1 × 10^6^ cells/mL. The suspension containing 9 million cells was then divided into three 5 mL polypropylene tubes (order number 55.526.006, Sarstedt, Nümbrecht, Germany), with 3 mL per tube. The tubes were placed in a silicone cup specially designed for this purpose and filled with water at the focus of the shockwaves [20]. The tubes were exposed to 1000 pulses, one after the other, at a frequency of 8 Hz and a power level of 20. The AdMSCs were then recultured together in a 75 cm^2^ cell culture flask with standard medium for eight hours. The standard FCS-containing medium was then removed by washing with a PBS solution. An additional two-day incubation period followed in standard medium with 10% Exo-FCS.

#### 2.3.3. Serum-Free Medium Cultivation

Cultivation in a serum-free medium was carried out using DMEM-LG and a 1% antibiotic solution. The medium contained 1% insulin–transferrin–selenite solution (ITS, order no. ITS-H, Capricorn, Germany) instead of FCS.

### 2.4. Collection and Concentration of EVs

To compare concentration methods for Western blot analysis and transmission electron microscopy (TEM), canine AdMSCs were cultured in 75 cm^2^ flasks in standard medium with Exo-FCS instead of normal FCS for three days. The resulting cell culture supernatant was then either ultracentrifuged or ultrafiltrated. For ultracentrifugation, the samples were first centrifuged for 10 min at 2700× *g*. Then, they were filtered through a 0.2 µm filter (order number 83.1826.001, Sarstedt, Germany) and loaded into the ultracentrifuge for one hour at 100,000× *g* using a 50.2 Ti rotor (Beckman Coulter, Krefeld, Germany). The resulting pellet was resuspended in 450 µL of PBS, corresponding to a 100× concentration. For ultrafiltration, the same procedure was followed, except a 100 kDa filter (Sartorius Vivaspin 15 mL Turbo, order no. 512-0565, VWR, Darmstadt, Germany) was used instead of ultracentrifugation at 1,000,000× *g*, and the supernatant was concentrated at 2700× *g* until it reached a 100× concentration (i.e., a volume of 450 µL).

For subsequent next-generation sequencing (NGS) analysis, the samples were concentrated 100× using ultracentrifugation. After treatment with standard medium, IL-1β, shock waves, and serum-free/ITS, the EV-containing medium was removed from a 75 cm^2^ bottle. This yielded a total of 15 mL of conditioned medium. The medium was ultracentrifuged as described, and 100 µL of the 100× concentrated solution was sent to a commercial provider for NGS analysis.

### 2.5. Nanoparticle Analysis

In accordance with MISEV guidelines [21], we examined the quality and quantity of the obtained particles for extracellular vesicles. First, nanoparticle tracking analysis (NTA) was performed to determine the particles’ size and concentration, regardless of quality. A total of six different canine donors provided samples for analysis (*n* = 6). Measurements were taken three times for 30 s each and averaged. Measurements were taken using a NanoSight LM10 (Malvern Instruments Ltd., Malvern, UK) with NTA 3.3 software, also from Malvern Instruments Ltd., UK. Details of the measurements can be found separately [22].

Additionally, transmission electron microscopy (Zeiss EM 109, Zeiss, Oberkochen, Germany) was performed on the obtained particles. For this purpose, the tetraspanins CD9 and CD81 were labeled with immunogold. The exact method is described elsewhere [22].

To exclude unwanted particles, such as nuclear particles (histones) and mitochondria (cytochrome C), and to characterize the EVs (ALIX and CD9) further, corresponding Western blots were performed [22]. The following antibodies were used: Ac-histones (clone E5, Santa Cruz Biotechnology, Heidelberg, Germany), cytochrome C (clone 7H8, Santa Cruz Biotechnology, Germany), CD9 (clone K41, Dianova, Hamburg, Germany), and CD81 (clone 5A6, Santa Cruz Biotechnology, Germany). In addition to the samples themselves, cell lysates were used as positive controls for histones and cytochrome C in the Western blot.

### 2.6. Nanoparticle Analysis—Statistics

A one-factorial analysis of variance (ANOVA) with repeated measurements was performed to determine the significance of the differences in the size and number of nanoparticles obtained. If significant differences among groups were found, a Tukey test was performed to identify the altered condition.

### 2.7. Nanoparticle Analysis—Transmission Electron Microscopy

To analyze the surface molecules CD9 and CD81, the previously obtained nanoparticles were fixed with 2% paraformaldehyde for further processing. Then, 5 µL of the solution was dropped onto glow-discharged, Formvar-carbon-coated nickel grids (TAAB Laboratories, Aldermaston, UK) and washed with PBS. The grids were incubated for three minutes at room temperature in a solution of 50 mM glycine and PBS. Additionally, permeabilization with 0.1% saponin was performed for membrane protein labeling. Next, the grids were labeled with antibodies and nanoparticles.

First, the primary antibodies CD9 (1:5, clone K41, Dianova, Germany) and CD81 (1:5, clone 5A6, Santa Cruz, Germany) were incubated, followed by a secondary antibody conjugated with 10 nm gold particles (EM.GMHL10, BBI Solutions, Freiburg im Breisgau, Germany, 1:5). For contrast, the grids were incubated for five minutes in a solution of 4% uranyl acetate and 0.15 M oxalic acid (pH 7.0; Sigma-Aldrich, Taufkirchen, Germany). Electron microscope images were taken with a Zeiss EM 109 at an accelerating voltage of 80 kV (Zeiss, Oberkochen, Germany).

### 2.8. NGS Analysis

After preparation, the 100× concentrates containing 100 µL were stored at −80 °C until all samples were collected. Three different donor dogs were used (*n* = 3). The samples were then sent for NGS analysis to determine the presence of miRNAs. Small RNA libraries were prepared using the efficient Small RNA-Seq Kit (GenXPro GmbH, Frankfurt, Germany) according to the manufacturer’s instructions. The small RNA molecules were ligated with 3′ and 5′ adapters containing TrueQuant UMIs. Then, they were reverse transcribed and PCR amplified with a minimal number of cycles. Finally, they were silica-bead purified. Sequencing was performed on an Illumina NextSeq instrument (Berlin, Germany) with 1 × 76 bp.

### 2.9. NGS Data Processing

Unprocessed sequencing reads were adapter-trimmed and quality-trimmed using Cutadapt (version 4.6 [23]) with the arguments “-e 0.1 -O 3 -q 20 -m 20 -n 8”. FastQC (0.11.9 [24]) was used to assess the quality of sequencing reads. Processed sequencing reads were mapped using Bowtie2 (2.4.4, [25]) on NCBI_cdna (with the arguments “--sensitive --local”) and mirna (with the arguments “--local --ma 1 --score-min L,0,0.9 --mp 1,1 --rdg 2,1 --rfg 2,1”) of UU_Cfam_GSD_1.0 (Canis lupus familiaris). The mapping was performed iteratively, meaning only the reads not mapping on the previous reference were mapped on the next one. Quantification of mapped reads to each transcript was performed using HTSeq (version 2.0.2, [26]) NCBI_cdna (with the arguments “-i transcript_id -r name -a 0 -m union” and strandedness “no”) and mirna (with the arguments “-i transcript_id -r name -a 0 -m union” and strandedness “no”). MultiQC (version 1.23 [27]) was used to create a single report visualizing the output from multiple tools across many samples, enabling global trends and biases to be quickly identified.

### 2.10. Differential Expression Analysis (DEA) Statistics

DEA was performed using DESeq2 (version 1.38 [28]). Only entries having at least a raw count of 5 in at least 2 samples were used in the DEA. Log2FoldChange values were shrunk using “ashr” [29]. DEA results with an FDR-adjusted *p*-value less than or equal to 0.05 and an absolute log2FoldChange greater than or equal to 1 were called significant.

## 3. Results

### 3.1. Cultivation and Characterization of Canine Adipose-Derived MSCs

**Figure 1:**
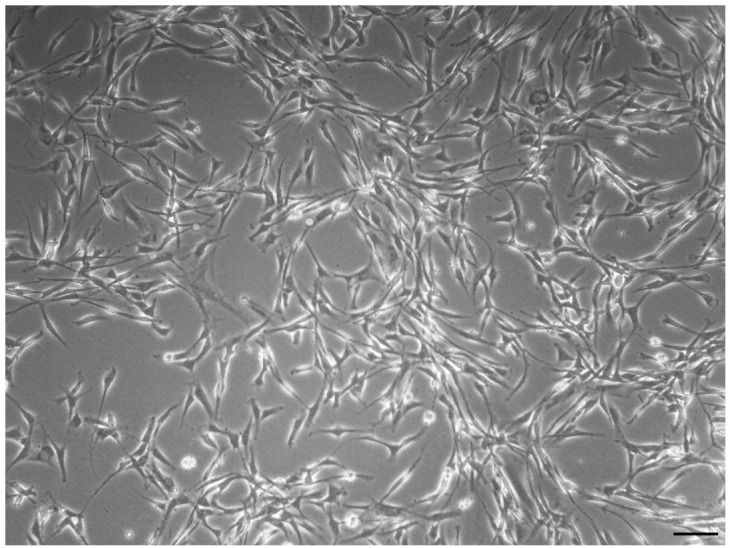
Typical appearance of adherent canine mesenchymal stem cells in a cell culture flask. Scale bar = 100 µm.

The AdMSCs that were isolated from adipose tissue had a typical spindle-shaped appearance and grew adherently on the cell culture flask (Figure 1).

**Figure 2:**
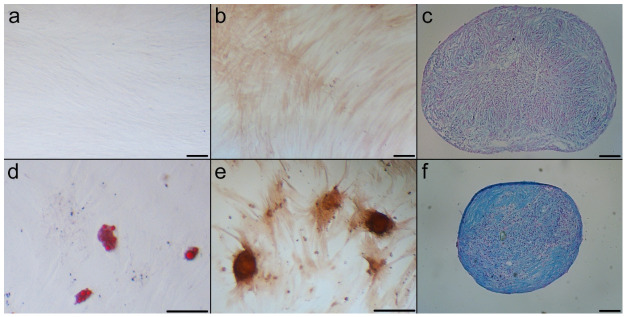
Differentiation into three lineages—adipogenic, osteogenic, and chondrogenic—is demonstrated. The upper row shows negative controls for adipogenic (**a**), osteogenic (**b**) and chondrogenic (**c**) differentiation in standard medium; the lower row shows AdMSCs cultured in adipogenic (**d**), osteogenic (**e**) and chondrogenic (**f**) differentiation medium. Indicator bar length = 100 µm.

After undergoing appropriate differentiation, clear signs of differentiation were evident (Figure 2).

**Figure 3:**
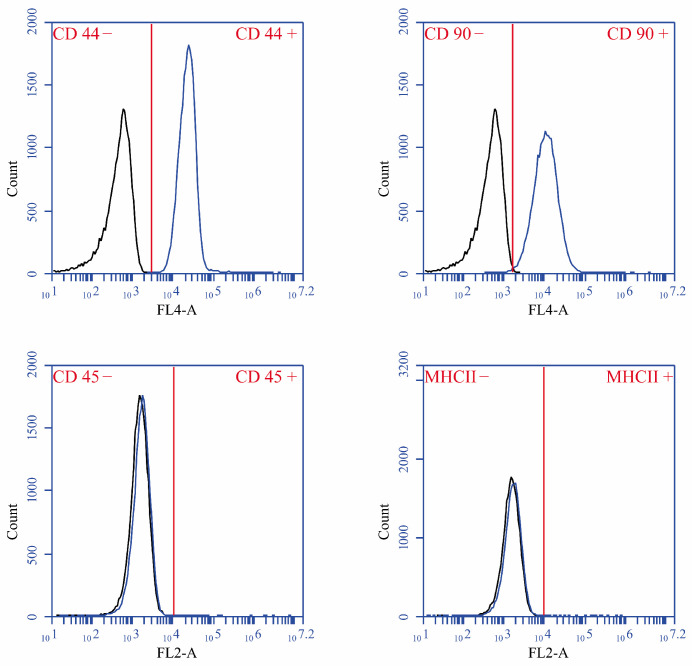
Flow cytometric results of the obtained stem cells. These are measurements of the surface molecules CD44, CD45, CD90, and MHCII for one donor, as an example. The black curve represents the negative control, and the blue line represents the measured sample. CD44 and CD90 surface markers are clearly positive, while CD45 and MHCII surface markers are not detectable.

After adipogenic differentiation, fat vacuoles were detected using Oil Red O staining. After osteogenic differentiation, nodule formation and calcium deposition were demonstrated using Alizarin Red staining. Finally, alcian blue staining revealed the distinct blue coloration of glycosaminoglycans—a component of cartilage—in histological sections of chondrogenically differentiated pellets. The respective negative controls with standard medium showed no evidence of spontaneous differentiation. A subsequent flow cytometric analysis clearly detected CD44 and CD90 surface markers, while CD45 and MHC II markers were not detected (Figure 3).

Accordingly, we meet the ISCT requirements for the presence of canine AdMSCs.

### 3.2. Collection and Concentration of EVs

**Figure 4:**
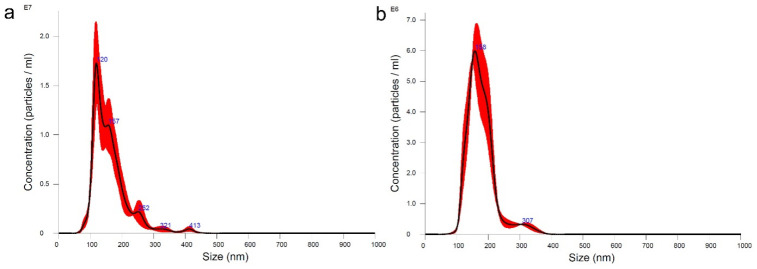
NTA measurements of cell culture supernatant from AdMSC cultures after ultracentrifugation (**a**) and ultrafiltration (**b**).

A total of six samples were examined using ultracentrifugation and ultrafiltration. Figure 4 shows an example of the size distribution after ultracentrifugation and ultrafiltration.

**Figure 5:**
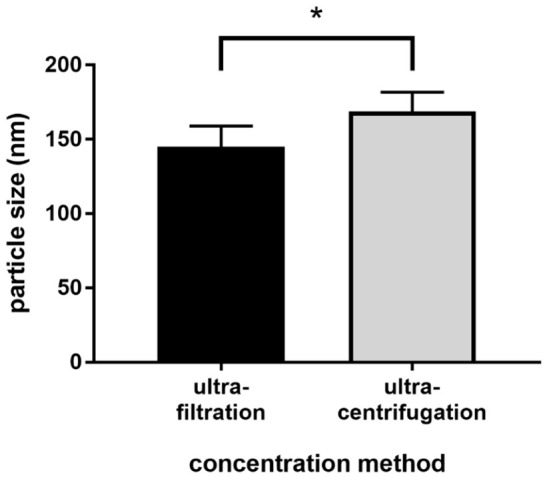
A comparative representation of the concentration methods of ultracentrifugation and ultrafiltration of EVs is presented. The mean particle size and standard deviation are shown. The asterisk indicates a significant difference (*p* = 0.040).

Regarding particle size, NTA analysis revealed that particles containing EVs were significantly larger after ultracentrifugation than after ultrafiltration (*p* = 0.040). After ultracentrifugation, the mean particle size was 168.7 ± 13 nm ($\bar{x}$ ± SD), whereas after ultrafiltration, the particles were on average 23.7 nm smaller at 145 ± 13.97 nm ($\bar{x}$ ± SD, Figure 5). The significant difference in size distribution appears to have been caused by a slight aggregation of the EVs following ultracentrifugation.

**Figure 6:**
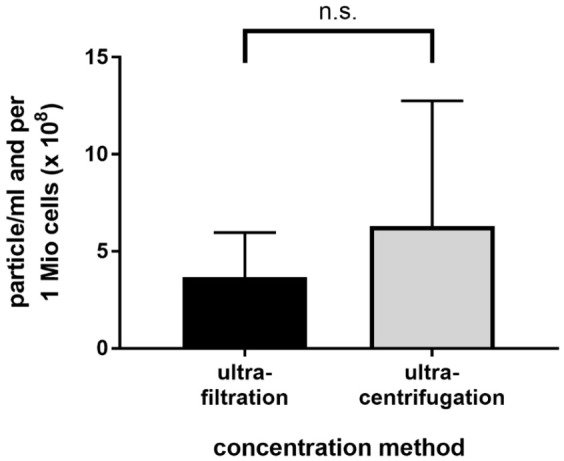
A comparative representation of the concentration methods of ultracentrifugation and ultrafiltration of EVs is presented. The mean particle concentration value and standard deviation are shown. The results were normalized based on cell count (per one million cells) in the cell culture bottles. The lack of significance is indicated by “n.s.” (not significant, *p* = 0.054) in the parentheses above.

No significant differences were found when comparing the concentration normalized to one million cells. Nevertheless, a trend is apparent (*p* = 0.054). According to this, the concentration after ultracentrifugation, at 6.29 ± 6.47 × 10^8^ particles/mL ($\bar{x}$ ± SD), is almost twice as high as after ultrafiltration, at 3.68 ± 2.29 × 10^8^ particles/mL ($\bar{x}$ ± SD, Figure 6).

### 3.3. Analysis of EVs

**Figure 7:**
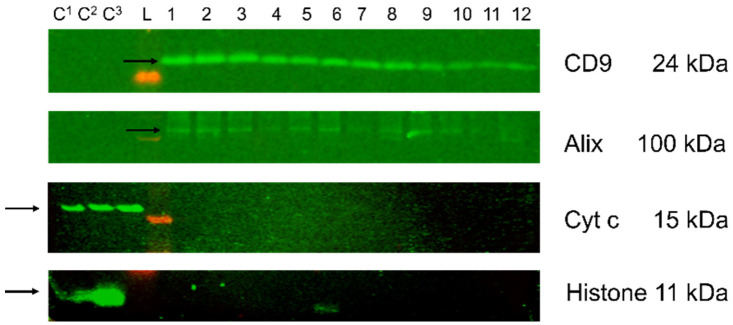
Western blot of positive markers (CD9, ALIX) and negative markers (Cyt C, histones) for extracellular vesicles. The size ladder is shown in red (L) and the samples in green (C1–C3, 1–12). Samples C1–C3 are controls of total cell lysates. Samples 1–6 are from donors 1–6 after ultracentrifugation; samples 7–12 are from donors 1–6 after ultrafiltration. The arrow on the left side indicates the relevant band height.

Western blotting was used to detect the relevant EV markers CD9 and ALIX in canine samples 1–6 after ultracentrifugation and in samples 7–12 after ultrafiltration. However, other cellular components, such as nuclear debris containing histones or mitochondria containing cytochrome C, were not detected. In contrast, positive detection was shown in canine total cell lysates (Figure 7).

**Figure 8:**
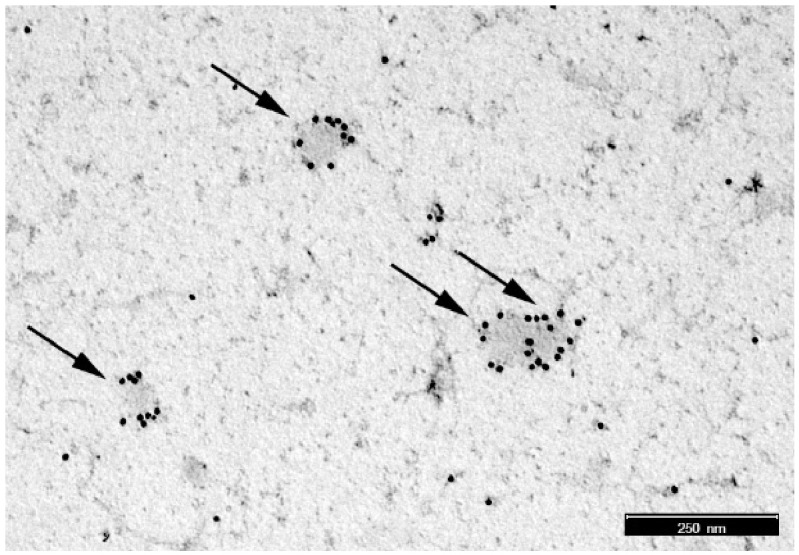
Transmission electron microscope images of canine EVs after immunogold labeling. The arrow points to EVs labeled with CD9.

**Figure 9:**
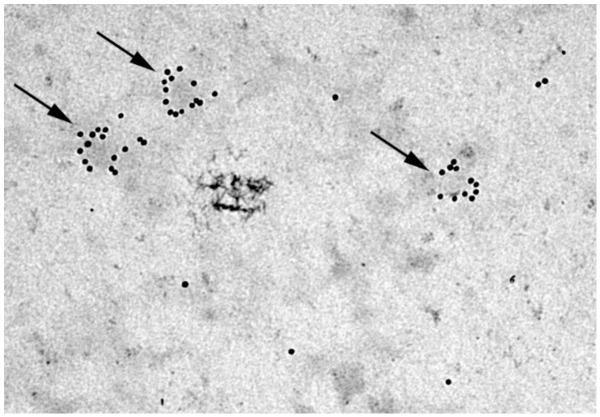
Transmission electron microscope images of canine EVs after immunogold labeling. The arrow points to EVs labeled with CD81.

Finally, transmission electron microscopy revealed that the tetraspanins CD9 and CD81 can specifically label particles (Figure 8 and Figure 9).

### 3.4. NGS Analysis of the Media Supernatant

**Figure 10:**
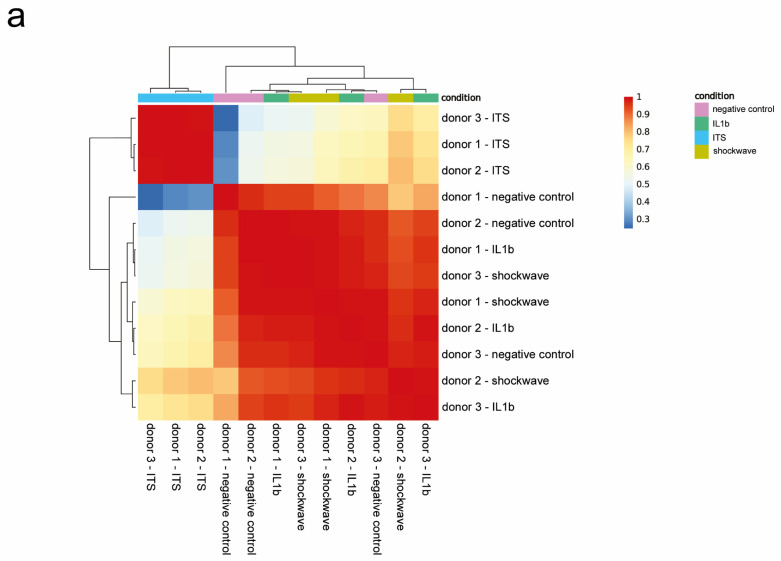

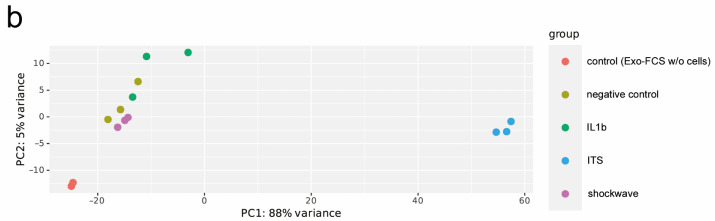
Unsupervised analysis of transcriptomic profiles across donors and experimental conditions. (**a**) Correlation heatmap of samples based on transcript expression values. (**b**) Principal component analysis (PCA) of the same transcriptome dataset. The control samples (Exo-FCS without cells) are from the EV-free FCS, which was used to rule out the presence of bovine microRNA.

After raw data processing, the average sequencing depth per sample was 610,760 reads. On average, 37.64% of reads mapped to NCBI_cdna and 0.54% of reads mapped to microRNAs (miRNAs). To investigate the overall structure of the transcriptomic data further, we performed an unsupervised correlation analysis and a principal component analysis (PCA) of the samples. The correlation heatmap revealed clustering based on experimental conditions. The ITS samples formed a distinct group. They were significantly different from the negative controls, IL-1β, and shockwave samples. The PCA confirmed this pattern. PC1 accounted for 88% of the total variance, primarily separating the ITS samples from the other conditions. The samples treated with shockwaves formed an additional distinct cluster. In contrast, the negative controls, which consisted of untreated mesenchymal stem cells (MSCs), and IL-1β-stimulated MSCs did not cluster very clearly. Together, these analyses suggest that the observed transcriptomic signatures are mostly determined by the experimental conditions, while variations between donors play a minor role (Figure 10).

A total of 209 different miRNAs were detected in the dog. In total, 84 of these were detected in the negative control, 69 after shockwave treatment, 90 after IL-1β treatment, and 201 after ITS cultivation (Supplement S1–S4). A comparison of statistically significant differences in miRNA expression showed that only 3 deviated significantly from the negative control after shockwave treatment (Table 1), 9 after IL-1β treatment (Table 2), and 83 after ITS cultivation (Table 3).

These results demonstrate that, following ITS treatment, all significant changes in expression increase. However, after shockwave and IL-1b treatments, one exception emerges: miR-199 is expressed at a lower level compared to the negative control without special treatment.

## 4. Discussion

Extracellular vesicles have become an important component of current research [30]. This is evident from the regular updates to the fundamental principles of extracellular particle research [31]. The important role of EVs in intercellular communication has only recently been recognized [32,33]. This is important to us because the field of intercellular communication of stem cells with their environment is still largely unclear. For example, in the case of mesenchymal stem cells used for therapeutic purposes, for example, characterizing and investigating extracellular vesicles would be valuable in order to better understand the mechanisms of action and possibly establish new cell-free treatment methods based on this knowledge [30]. To this end, it is important to enable the clear detection of EVs from the respective species. We have already successfully achieved this for horses [20,22]. However, the lack of suitable antibodies has been a significant obstacle in providing evidence that aligns with the MISEV guidelines and enables the clear identification of EVs from dogs. Only a few have met some of the MISEV guidelines so far, such as demonstrating the presence of CD81 or CD9 in a Western blot [34], or CD63 and CD9 [35]. Dogs are important candidates for investigating the mode of action and potential of EV-based therapeutics in veterinary medicine because they regularly suffer from osteoarthritis [6]. Common risk factors include age, genetic predisposition (breed predisposition), trauma, and obesity [6]. Some of these factors are highly transferable to osteoarthritis in humans [7], which is why we hope that the results of research into osteoarthritis in dogs will provide insight into human osteoarthritis and possible treatment strategies.

We found suitable antibodies that enable us to identify EVs from dogs. The tetraspanins CD9 and CD81 are important markers that can be used for Western blotting. However, positive controls contained cellular components such as nuclear particles (detected using antibodies against histones) and mitochondria (detected using antibodies against cytochrome C), which were not present in samples with purified extracellular vesicles. Additional investigations with immunogold labeling of the surface molecules CD81 and CD9 (tetraspanins) provided visual evidence of how gold particles bind to the secondary antibody and EVs. NTA investigations clearly demonstrated the success of extracting and concentrating the nanoparticles, consistent with our previous investigations in horses. Ultracentrifugation was shown to be superior to ultrafiltration in terms of EV yield. However, this method results in significantly larger vesicles, which we attribute to clumping or fusion of the particles due to the high g-forces.

After characterizing the canine EVs, we analyzed the associated miRNA. We chose microRNA analysis because it provides valuable insights into the mode of communication and can identify markers for disease presence or staging [36], as well as potential therapeutic candidates. Anti-miRNAs were also used to clinically test candidates for reducing disease-promoting miRNAs [37]. We had previously conducted and published a similar study on equine EVs [20].

For this study, we sent a comprehensive analysis of the miRNAs present in the canine EVs to a commercial service provider. We created four different sample sets, modifying the conditions for producing EVs as follows: First, we compared EV-depleted FCS to an FCS-free cell culture medium with an insulin–transferrin–selenium (ITS) solution. FCS naturally contains extracellular vesicles, which should be excluded from subsequent analyses. To achieve this, EV-depleted FCS or a full cell culture medium that contains all the necessary factors to ensure continued cell growth and survival must be used instead of FCS. In this context, we demonstrated that the expression of 83 different miRNAs was significantly altered, particularly compared to EV-depleted FCS. This result is noteworthy because it underscores the critical role of the FCS replacement supplement. This factor should be considered in future studies, especially when using ITS.

The other two conditions to which the canine mesenchymal stem cells were exposed were shock waves and the addition of IL-1β. Using shock waves to treat osteoarthritis is a well-established adjunctive therapy. It is popular because it is noninvasive and has been shown to improve outcomes in vitro [38] and in vivo [39]. By contrast, we used IL-1β to model MSC damage. Previous studies have shown that IL-1β is a suitable inflammatory mediator for simulating OA [40,41,42] and can serve as a priming agent for MSCs [42,43]. This priming has been shown to positively affect joint damage caused by the excessive proliferation of fibroblast-like synoviocytes, which play a key role in OA [44].

Shock waves were used to influence the cells as a therapeutic mechanical stimulus, and the addition of IL-1β mimicked an inflammatory process. Surprisingly, compared to the negative control without stimulation, only a few significant differences were found. Based on our previous study with EVs obtained from horses under comparable conditions, we expected to find more significant differences. Only eight miRNAs were significantly higher after the administration of IL-1β, and one was lower. After shockwave stimulation, only two miRNAs were significantly higher, and one was lower. In both cases, the same miRNA, CFA-mir-199, was reduced.

In a study by Lee et al. [45], TNF-α and IFN-γ were added to stimulate canine MSCs after EVs were isolated and their miRNAs were analyzed. Only 11 miRNAs were found to show significantly altered expression in this study. Two of these, miR-16 and miR-502, increased significantly. However, unlike the aforementioned study, our studies only detected an increase in miR-16 following IL-1β and ITS treatment. The remaining miRNAs decreased significantly. Five different miRNAs overlapped with our results, but they either increased in our study or changed significantly only after ITS treatment (miR-7c, -7f, -7g, and -103).

Further examination of the individual miRNAs is difficult due to contradictory information in the literature. For example, there is conflicting information regarding miR-24. We demonstrated that miR-24 expression increases significantly after shockwave treatment, IL-1β stimulation, and ITS conditioning. This increase could represent a stress-related response to various treatment options [46]. It is known that oxidative stress leads to increased expression of miR-24, which inhibits DNA repair and results in cell senescence [47]. However, another study showed that miR-24 has a protective effect on damaged cardiomyocytes, improving cell viability and protecting against apoptosis [48]. In another context, miR-24 is described as being essential for the differentiation of hematopoietic stem cells into blood cells [49], as well as for the differentiation of embryonic stem cells, by blocking pluripotency factors such as OCT4 [50]. Without further experimentation, it is difficult to assess the specific function of this microRNA, particularly with regard to gaining a deeper understanding of the harmful effects on our model and considering microRNAs as a therapeutic element.

The expression of miR-361 increased significantly after shockwave and ITS treatments. This has been described in the literature as an important factor in epithelial–mesenchymal transition, which impairs control of renewal processes [51]. However, studies on tumor development have shown that increased expression of miR-361 actually reduces epithelial–mesenchymal transition. In tumors, this results in reduced invasive growth and lower metastasis [51]. In terms of stem cell mobilization, increased expression of miR-361 could negatively impact stem cell activity.

Reduced expression of miR-199, as demonstrated following stimulation with IL-1β and shockwaves, has been described as promoting differentiation into cardiomyocytes [52] and adipogenic differentiation [53]. Conversely, increased expression of miR-199a and miR-199b (which belong to the miR-199 family) has been shown to promote the chondrogenic differentiation of mesenchymal stem cells [54,55,56]. Based on miR-199 alone, one could argue that there is evidence of its role in an IL-1β-based osteoarthritis lesion model. However, further evidence is certainly needed to confirm this hypothesis.

It should be emphasized that it is not possible to discuss all expressed miRNAs because doing so would exceed the scope of this paper. Future studies must analyze and investigate these data further in experiments to determine their actual influence on the development and treatment of osteoarthritis.

At this point, we would also like to address the limitations of this study. Due to the limitations of NGS analysis, it was not possible to include a larger number of stem cell donors in our study. To ensure the highest possible standardization of results, even with a small sample size, we outsourced the NGS analysis to a specialized external service provider. A correlation heatmap and a principal component analysis (PCA) were performed to determine whether the results were due to a treatment effect or differences among the donors, because the sample size was small. A strong correlation for the ITS treatment is evident. Shockwave therapy also showed a trend, although the difference compared to the untreated control group and the IL-1β treatment group was not particularly clear or significant.

Additionally, we did not anticipate such a high number of altered miRNAs with ITS treatment, especially compared to our other stimulation methods. This demonstrates, in hindsight, the essential nature of a standardized, gentle method of EV collection.

A functional analysis of the miRNAs identified via NGS is urgently needed to validate their effects on other cell types, such as mesenchymal stem cells (MSCs) or chondrocytes. This would pave the way for clinical applications. This was not possible within the scope of this study, but will be addressed in future studies.

## 5. Conclusions

We successfully purified EVs from canine MSCs using the described methods. We unambiguously confirmed the identity of the EVs with appropriate antibodies while excluding particulate contaminants that might otherwise be misidentified as EVs. NGS revealed that the expression of several miRNAs was significantly altered by different stimulation conditions. In particular, the significant effects observed following FCS-free culture with ITS provide important insights into how culture conditions influence miRNA profiles associated with EVs. These effects also underscore the need for strict standardization in research on extracellular vesicles.

Due to the limited number of donors, we also evaluated the global sample structure using a correlation heatmap and principal component analysis (PCA) to distinguish treatment effects from donor-related variation. These analyses showed that the ITS-treated samples formed the clearest and most distinct cluster, followed by the shock-wave-treated samples. The negative control and IL-1β groups, on the other hand, clustered less distinctly and showed more overlap. Nevertheless, these results suggest that the observed changes in the transcriptome are primarily due to the experimental conditions, while donor-related variations appear to play a minor role.

The obtained miRNA data provide a basis for the targeted functional validation of individual candidate miRNAs in assays involving MSCs or chondrocytes to investigate their role in inflammatory and regenerative processes. Thus, these findings may contribute to veterinary medicine and serve as a translational model for human regenerative research.

## Figures and Tables

**Table 1:** List of gene expressions significantly altered by shockwave treatment.

Name	log2fc
cfa-miR-361	5.05
cfa-miR-24	2.96
cfa-miR-199	−2.20

The name of the microRNA (miRNA) and the log_2_ fold change in gene expression compared to the negative control (untreated AdMSC) are shown. The *p*-values are less than 0.05; exact values can be found in the Supplementary Materials.

**Table 2:** List of gene expressions significantly altered by IL-1β.

Name	log2fc
cfa-miR-451	9.94
cfa-miR-486-3p	5.64
cfa-miR-486	4.95
cfa-miR-185	3.88
cfa-miR-223	3.82
cfa-miR-24	3.31
cfa-miR-101	2.38
cfa-miR-16	1.73
cfa-miR-199	−2.05

The name of the microRNA (miRNA) and the log_2_ fold change in gene expression compared to the negative control (untreated AdMSC) are shown. The *p*-values are less than 0.05; exact values can be found in the Supplementary Materials.

**Table 3:** List of significantly altered gene expressions after serum-free/ITS conditions.

Name	log2fc	Name	log2fc	Name	log2fc
cfa-miR-365	8.72	cfa-let-7f	6.17	cfa-miR-20a	4.62
cfa-let-7c	8.28	cfa-miR-331	6.11	cfa-miR-340	4.60
cfa-miR-15b	8.21	cfa-miR-30b	6.11	cfa-miR-125b	4.59
cfa-miR-29b	8.12	cfa-miR-148b	6.04	cfa-miR-140	4.58
cfa-miR-374a	8.07	cfa-miR-26b	6.03	cfa-miR-432	4.57
cfa-miR-22	7.98	cfa-let-7b	5.96	cfa-miR-532	4.56
cfa-let-7e	7.92	cfa-miR-374b	5.95	cfa-miR-99a	4.56
cfa-miR-152	7.78	cfa-let-7g	5.94	cfa-miR-25	4.54
cfa-miR-98	7.63	cfa-miR-103	5.79	cfa-miR-181d	4.45
cfa-miR-379	7.57	cfa-miR-1271	5.76	cfa-miR-193a	4.38
cfa-miR-221	7.51	cfa-miR-127	5.75	cfa-miR-30a	4.32
cfa-miR-151	7.48	cfa-miR-186	5.71	cfa-miR-574	4.25
cfa-miR-145	7.17	cfa-miR-185	5.70	cfa-miR-92a	4.17
cfa-let-7a	7.13	cfa-miR-125a	5.64	cfa-miR-148a	4.09
cfa-miR-24	6.92	cfa-miR-378	5.62	cfa-miR-320	4.06
cfa-miR-196b	6.86	cfa-miR-30e	5.57	cfa-miR-101	3.98
cfa-miR-383	6.69	cfa-miR-494	5.53	cfa-miR-27b	3.94
cfa-miR-30c	6.57	cfa-miR-455	5.50	cfa-miR-16	3.91
cfa-miR-28	6.52	cfa-miR-8859a	5.49	cfa-miR-214	3.85
cfa-miR-361	6.43	cfa-miR-26a	5.42	cfa-miR-106b	3.76
cfa-miR-1307	6.39	cfa-miR-30d	5.35	cfa-miR-19a	3.46
cfa-miR-8859b	6.37	cfa-miR-191	5.25	cfa-miR-196a	3.41
cfa-miR-590	6.37	cfa-miR-21	5.13	cfa-miR-34a	3.21
cfa-miR-93	6.28	cfa-miR-29a	5.04	cfa-miR-423a	3.14
cfa-miR-155	6.25	cfa-miR-143	4.80	cfa-miR-197	2.85
cfa-miR-222	6.21	cfa-miR-10a	4.71	cfa-miR-15a	2.72
cfa-miR-7	6.20	cfa-miR-99b	4.71	cfa-miR-19b	2.62
cfa-miR-10b	6.19	cfa-miR-199	4.63		

The name of the microRNA (miRNA) and the log_2_ fold change in gene expression compared to the negative control (untreated AdMSC) are shown. The *p*-values are less than 0.05; exact values can be found in the Supplementary Materials.

## Data Availability

Data not included in this manuscript are available upon reasonable request.

## Supplementary Materials

The following supporting information can be downloaded at: https://www.mdpi.com/article/10.3390/ani16121872/s1, Raw data of expressed miRNA and significant differential expressions (txt-File tab stop separated): Supplement S1: Expressed_miRNA; Supplement S2: NK-vs-serumfree_ITS; Supplement S3: NK-vs-IL_1b_significant; Supplement S4: NK-vs-shockwave_significant. The raw data from our NGS analysis in FASTQ format can be found at: doi.org/10.22029/JLUPUB-20939.

## Abbreviations

The following abbreviations are used in this manuscript:

AdMSC	adipose-derived mesenchymal stem cell
CD	cluster of differentiation
DEA	differential expression analysis
DMEM-LG	Dulbecco’s minimal essential medium—low glucose
EV	extracellular vesicle
Exo-FCS	exosome-depleted fetal calf serum
FCS	fetal calf serum
IL-1b	Interleukin 1b
ISCT	International Society for Cellular Therapy
ITS	insulin–transferrin–selenite
MHC	major histocompatibility complex
miRNA	microRNA
MISEV	minimal information for studies of extracellular vesicles
MSC	mesenchymal stem cell
NGS	next-generation sequencing
NTA	nanotracking analysis
PBS	phosphate buffered saline
PCA	Principal component analysis
RNA	ribonucleic acid
